# Further We Travel the Faster We Go

**DOI:** 10.1371/journal.pone.0148913

**Published:** 2016-02-10

**Authors:** Levente Varga, András Kovács, Géza Tóth, István Papp, Zoltán Néda

**Affiliations:** 1 Babeş-Bolyai University, Department of Physics, Cluj-Napoca, Romania; 2 Edutus College, Department of Trade and Marketing, Tatabánya, Hungary; 3 Hungarian Central Statistical Office, Budapest, Hungary; Beihang University, CHINA

## Abstract

The average travelling speed increases in a nontrivial manner with the travel distance. This leads to scaling-like relations on quite extended spatial scales, for all mobility modes taken together and also for a given mobility mode in part. We offer a wide range of experimental results, investigating and quantifying this universal effect and its measurable causes. The increasing travelling speed with the travel distance arises from the combined effects of: choosing the most appropriate travelling mode; the structure of the travel networks; the travel times lost in the main hubs, starting or target cities; and the speed limit of roads and vehicles.

## Introduction

Understanding universal laws governing human mobility is in the focus of many recent studies [1–5]. Data obtained from electronic databases, such as GPS [6], mobile phone [5, 7–9] and banknote tracking [1], time-table [10] or commuting data [3, 4], location-based social services and socialization sites [11, 12], revealed phenomenological laws and allowed for testing many mobility models [3–6, 13–16]. The main problems investigated so far, concerned mobility patterns [5–7] and mobility fluxes inside and/or between settlements [3–6, 10, 12, 14–16]. Apart of the obvious fundamental interest, such studies have immediate economic and social applications. They allow for a better traffic optimization and planning [10, 17–19], policies for preventing spreading of epidemics [20, 21], and also help for developing businesses around human mobility. Here, we study another aspect of human mobility, revealing a general pattern relating the average speed of the mobility to the travel distance.

From our everyday experience we have learned, that the travelling time does not scale linearly with the travel distance [5, 10, 17, 22–24]. It might take several hours to travel at distances of a few hundreds of miles, but not significantly more to travel at the opposite side of the Earth. This is of course due to the fact that for smaller distances we use slower transportation modes, cars or commuting trains with many stops, while at larger scale we travel faster by airplanes or high-speed trains. Similar observations can be made for urban travel. In cities, travels at shorter distances are dominated by bus travel or walking, while at larger distances faster modes (trains or metro) are used. We recall here the surprising and extreme result, according to which in some major cities (like Boston or Lisbon) the commuting time is roughly independent of the commuting distance [5]. Analysing the British public transportation system, it has been also proved [10] that in general urban travels are multimodal with significant connection times between different modes. The combined effects of these modes and waiting times leads to a sub-linear dependence of the travelling time as a function of the travel distance [10].

Surprisingly however, even if we do focus on the same travelling mode we still find an increasing effective speed with distance. For example, when driving from one city to the other, we notice that the driving time increases sub-linearly with the driving distance. The reason behind this is more elaborate and involves several effects. For larger distances we use instead of country roads highways, where the connection is straighter, they are less intersections, less emergency stops and the speed limit is higher. Another reason for this is that usually much of the travelling time is spent for getting out and in the city, reducing drastically the average travelling speed for short travels. For air travel, the situation is similar. Smaller distances are served with smaller planes, travelling at lower altitude, where the cruising speed is smaller. Much of the travelling time is used for the takeoff, landing and parking which is roughly the same independently of the travel distance, reducing drastically the average speed of short flights. These combined effects leads to a higher average travelling speed for the long intercontinental flights, in comparison with the shorter flights.

Although the above discussed phenomena are quite universal, well known and affects our everyday life, up to our knowledge there has been no attempt to quantify them. Using several electronic databases and GPS tracking, here we explore in more detail these effects and look for their measurable causes.

## Results and Discussion

**Fig 1:**
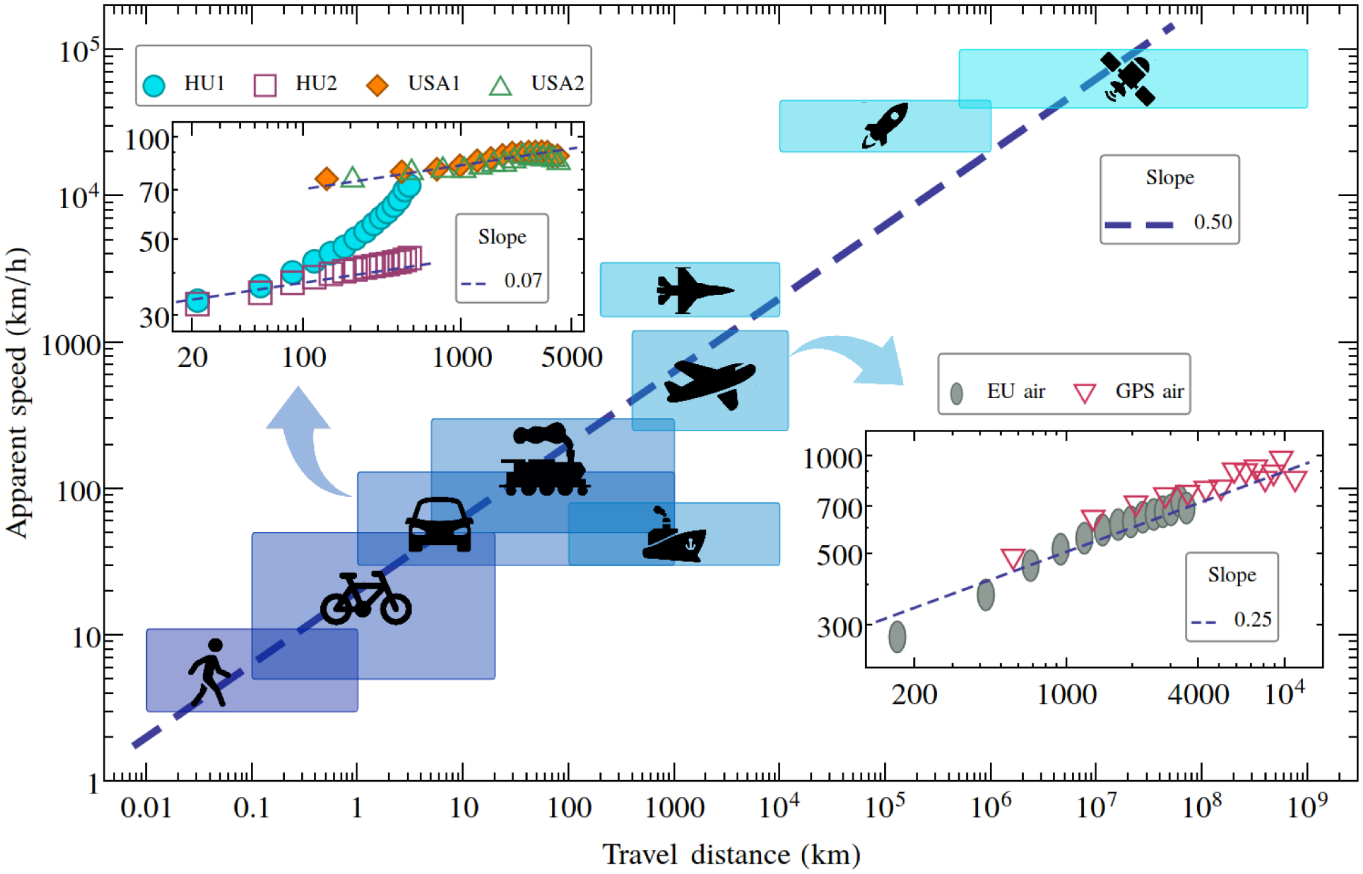
Velocity and distance scales of human travel. The apparent travel speed (estimated as the travelled distance on the geodesic line divided by the travel time) as a function of the travel distance. Boxes indicate intervals for different travelling modes. The two inset figures present some averaged results broken down on the two most popular travelling modes: car and air travel. The dashed lines in these insets indicates a power-law trend with the specified exponent. For the source of the data, and averaging method, see the Methods section. Dashed lines with different slopes are not fitting results, they indicate a power-law trends with the specified exponents only for guiding the eyes. Please note the logarithmic axes.

First, let us roughly estimate the distance and velocity scales for all human travelling modes, ranging from pedestrian walking up to cosmic travel. Using a logarithmic scale, on Fig 1 we indicate with boxes the characteristic spatial and velocity intervals for each travelling mode. As a first striking result we see, that the boxes are following a power-law like trend with an average exponent around 0.5 on an extended distance and velocity scale. If we zoom inside a box (consider only one specific travelling mode) and study averaged velocities as a function of the travelling distance, a similar, monotonically increasing trend is found. In the insets of Fig 1 we present results on car and air travels separately.

We can learn from these results that in case one considers roads of the same rank (HU2, USA1, USA2: roads with similar speed limits), a trend resembling a power-law is obtained with similar scaling exponents (approx. 0.07). In case the travel on all major roads (national roads and highways) are allowed (HU1) the results do not scale and the monotonically increasing trend is much steeper. For air travel we used only data on direct flights between airports (see the section Methods) and the results suggests again a monotonic increase of the apparent speed as a function of the travel distance. Although the results do not indicate a perfect scaling, if one would force a power-law on the bin-averaged data, an exponent around 0.25 would be a reasonable approximation for the trend. The results presented in Fig 1 suggest, that the increase in the travelling speed becomes more and more pronounced once more diverse data are used. The observation that for a given travelling mode, the apparent speed is increasing with the geodesic distance of the travel, has several measurable causes.

**Fig 2:**
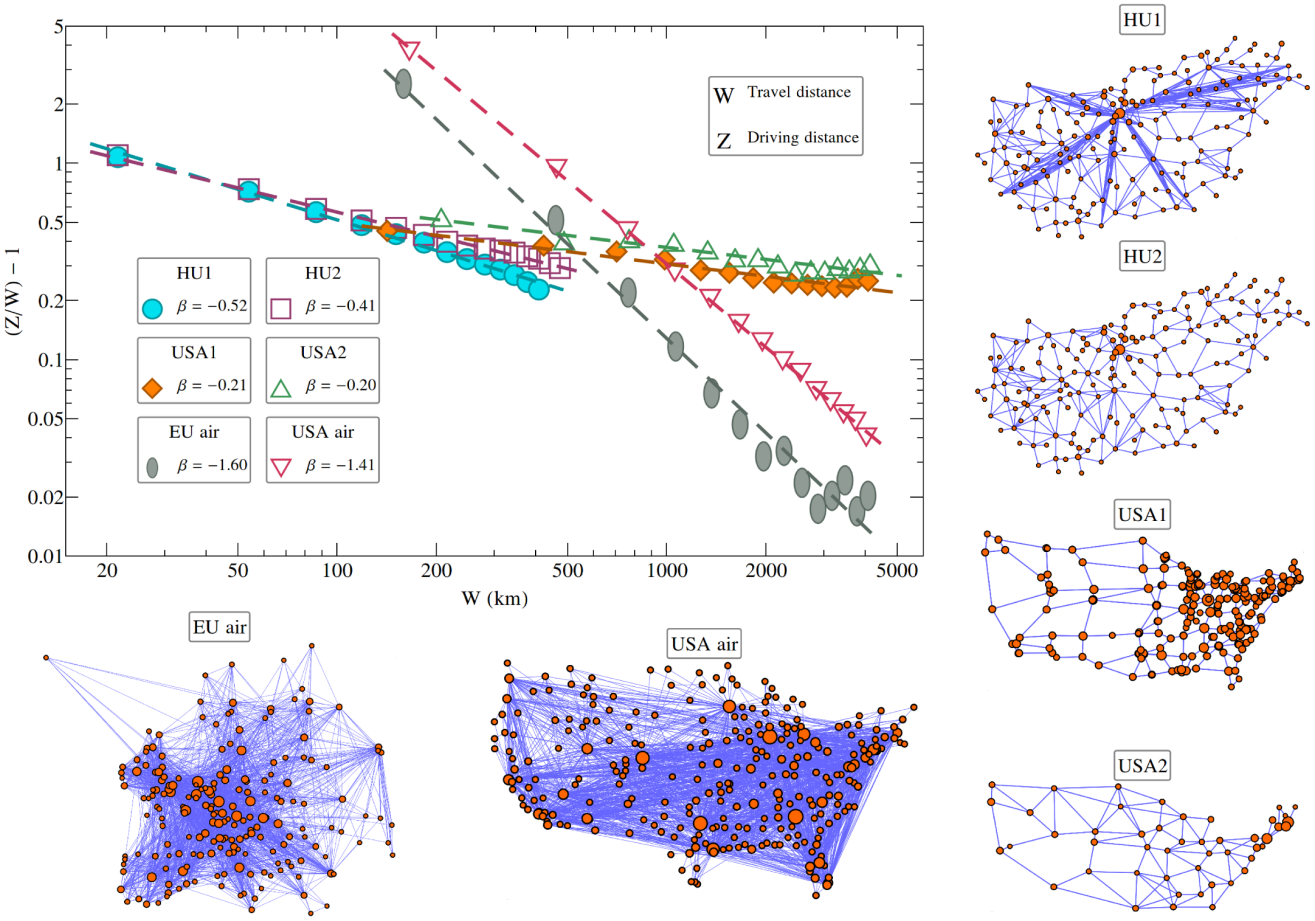
Topology of travel networks. Scaling between the driving distance (*z*) and geodesic distance (*w*) on different travel networks. The curves suggest the $\frac{z}{w}-1=C\cdot w^{-\beta }$ relation, with the parameters given in Table 1. The figure illustrates also the topological structure of some human transportation networks used in the present study (see the Methods section). Please note the logarithmic axes.

A first, quite evident cause, results from the structure of the networks on which the mobility is realized. Nodes are in general not connected through straight-line roads, and usually there are no direct paths between each of them [25]. In order to go from one node to another, travellers follows a path in the graph with a minimal road-length (the length of this path is named here as *driving distance* and denoted as *z*). As the *w* *travel distance* (geodesic distance) increases, the driving distance approaches *w*. Analysing the topology of some transportation networks used in this study (Fig 2) we find that these two distances are in average related to each other in form of a scaling relation:

$$\begin{array}{c} \frac{z}{w}-1=C\cdot w^{-\beta } \end{array}$$ (1)

The goodness of the above scaling is supported by the *R*^2^ > 0.9 parameters obtained for the power-law fits, summarized in Table 1. The trends for the air travel network and road network clearly separates through the fitted *β* exponents. For air travel we find *β* ≈ 1.4−1.5 and for the road travel *β* ≈ 0.2−0.3. This means that in the case of air travel the value of *z* converges more quickly to *w* than in the case of the road-networks. According to this, for a hypothetically constant *u* travelling speed along the roads (or cruising speed for the airplanes) and no time-lapse at the nodes, one would get an increasing trend for the apparent speed, *v*, as a function of *w*:

$$\begin{array}{c} v=\frac{w}{t}=\frac{w}{z}\cdot u=\frac{u}{1+C\cdot w^{-\beta }} \end{array}$$ (2)

**Table 1:** Fitting parameters for the data in Fig 2.

	HU1	HU2	USA1	USA2	EU air	USA air
*β*	0.52	0.41	0.21	0.20	1.60	1.41
*C*	1.73	1.32	0.27	0.42	8.97	8.55
**R**^**2**^	0.99	0.99	0.93	0.90	0.98	0.99

Fitting parameters for different experimental data, considering the Eq (1) approximation. The *R*^2^ ≥ 0.9 values indicates that the scaling hypothesis Eq (1) is justified. For the description of the experimental data see the Methods section.

**Fig 3:**
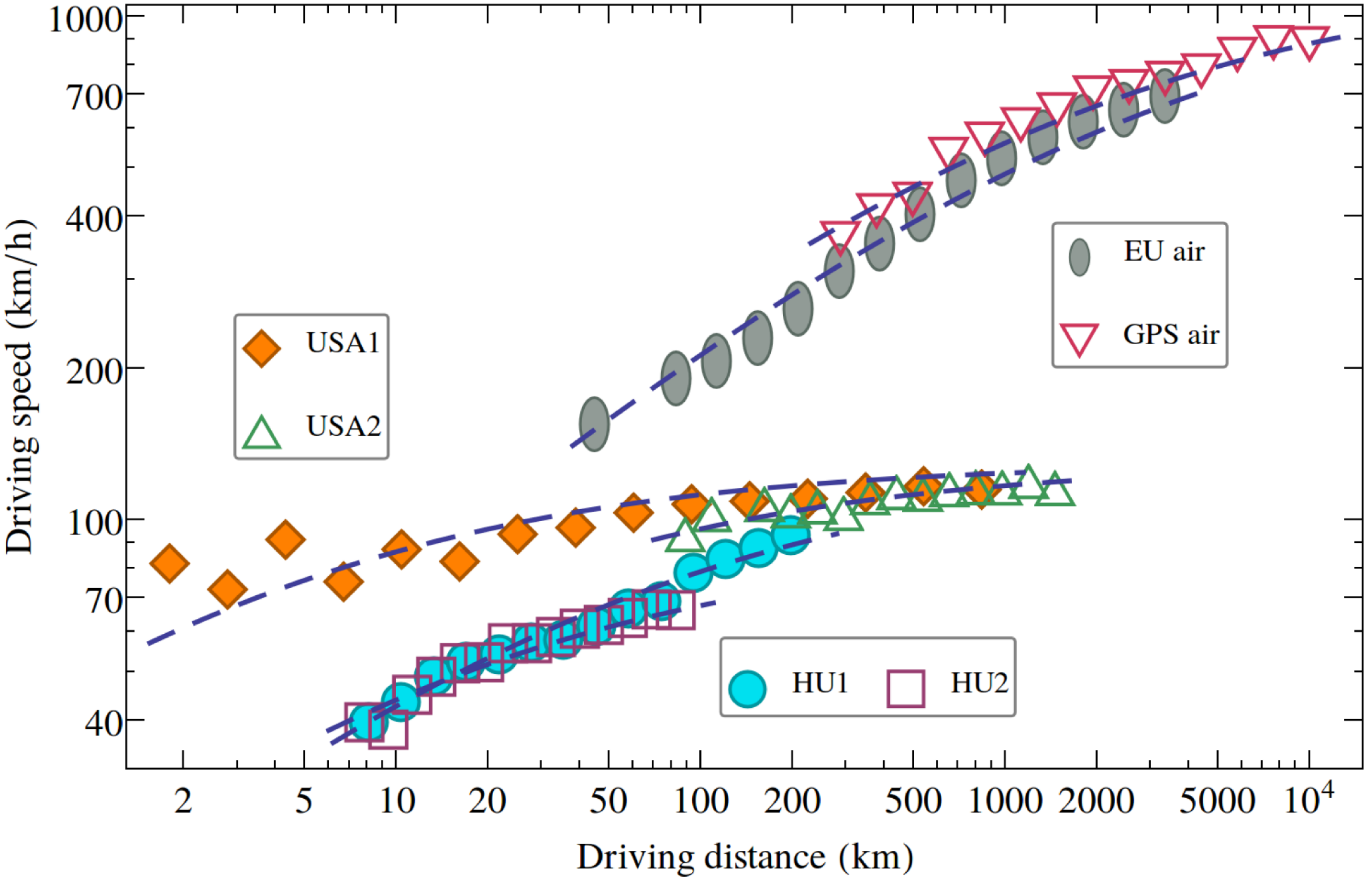
Driving speed increases with driving distance. Results for different travelling modes and networks. The data can be approximated with the formula suggested in Eq (3) using *α* = 0.5 (the fits indicated with dashed lines in this figure). Taking however *α* also as a free fitting parameter the fits are even more performant, as one can observe this in Table 2. Please note the logarithmic axes.

This simple geometric argument derived from the topology of the mobility networks is not sufficient however to fully understand the increase of the apparent speed as a function of the travel distance. In Fig 1, in the case of air travel we considered only direct (and presumably straight-line) flights between airports assuming *z* = *w*, and we still observed the increase of the apparent speed as a function of the travel distance. The same result holds if we consider only direct connections between the nodes in the road-network. In such case the average *cruising speed*, or *driving speed* on a direct link, defined as $u=\frac{z}{t}$, is also increasing with the driving distance, *z*. Results in such sense are plotted in Fig 3.

The increasing trend of the cruising speed as a function of the driving distance on a direct segment, might have again multiple causes. First, one can argue that for each link both the origin and target nodes induce delays. In a transportation network the nodes are cities, in which the complexity of the traffic obviously generate delays. Longer links have the origin in larger cities (longer intercontinental flights, larger highway segments), and larger cities induce larger delays. Secondly, on each linear segment delays are present outside the nodes too. This is due to traffic conditions that deviate from the ideal. For longer segments longer delays are expectable, however the increase is not necessarily linear. The combined effect of the two types of delay will generate the increasing trend of the cruising speed. If we assume that on a given segment there is a limiting cruising speed, *u*_0_, and the delays are increasing as a power-law with the, *z*, length of the segment: *t*_*delay*_ = *K*⋅*z*^*α*^, we get:

$$\begin{array}{c} u=\frac{z}{\left(\frac{z}{u_{0}}\right)+t_{delay}}=\frac{1}{\left(\frac{1}{u_{0}}\right)+K\cdot z^{\alpha -1}} \end{array}$$ (3)

For *α* < 1, we get an increasing trend as a function of *z*. On Fig 3 we show that the fits of the form Eq (3) are qualitatively good for all the experimental data on car and air travel even when we fix the *α* = 0.5 exponent. The fits presented by the dashed lines on Fig 3 were obtained by fixing also the *u*_0_ speed limit. We considered the *u*_0_ = 90 km/h speed limit for the HU2 (national road) data, *u*_0_ = 130 km/h for highways and interstates (HU1, USA1, USA2) and *u*_0_ = 1200 km/h ≈1 Mach for the air travel. The results suggest thus (see Fig 3) a universal convergence law to the *u*_0_ limiting speed value. A better quality fit for all the data can be obtained when considering the *α* exponent also as a fitting parameter. The fit parameters and the goodness of the fit (characterized by *R*^2^) are summarized in Table 2 both for the case when *α* was fixed or considered as a free fitting parameter. We learn from this data that for *α* = 0.5, Eq (3) describes only qualitatively the experimental data. On the other hand, when *α* is also a free fitting parameter, Eq (3) provides also a quantitatively correct fit.

**Table 2:** Fitting the experimental data with Eq (3).

	HU1	HU2	USA1	USA2	EU air	GPS air
$\bar{u_{0}}$(km/h)	130	90	130	130	1200	1200
*α*	0.49	0.44	0.69	0.58	0.44	0.43
*K*	-2.94	-3.13	-4.91	-4.07	-2.94	-2.99
**R**^**2**^	0.97	0.96	0.90	0.87	0.99	0.96
$\bar{\alpha }$	0.50	0.50	0.50	0.50	0.50	0.50
$\bar{u_{0}}$(km/h)	130	90	130	130	1200	1200
*K*	-2.98	-3.33	-4.20	-3.60	-3.28	-3.51
**R**^**2**^	0.97	0.95	0.54	0.84	0.98	0.95

Fitting parameters for the experimental data, considering the approximation given by Eq (3). Two different calculations are presented. In the upper part of the table we present the fit results when both the *α* and *K* parameters are considered as free fitting parameters. In the bottom part of the table we present the fit results when only *K* is considered as free fitting parameter. In both cases the fixed parameters used in fitting Eq (3) are overlined.

These results suggests that for a direct link in a given transportation network, the cruising speed and consequently the apparent travelling speed is increasing as a function of the length of the link, approaching the *u*_0_ speed limit only for infinitely long segments.

Finally, the limiting speed value also increases with the length of the road- or flight-segment. As the road-segment is longer, the speed limit is usually increased. Highways have longer segments and increased speed limit comparable with national roads. On longer air travel segments, usually faster airplanes are cruising, and similar effect is true for rail-travel.

In conclusion, our quantitative results support the starting hypothesis according to which further we travel the faster we go. Increase of the averaged apparent speed as a function of the distance follows in a first approximation a power-law like trend, both for all the travelling modes taken together, and for one selected mode in part. The cause for this increased travelling speed is multiple. We have shown here that both the network structure of the road topology, the slowdown effects of the main hubs (cities), travel conditions deviating from the ideal and the increasing speed limit for larger travel-segments are contributing together for generating this universal effect.

Planning related to travelling modes, travelling time and their infrastructure networks has to take into account this simple empirical law. In order to increase the apparent speed one must first optimize the road-network (or air-connection networks), so that the characteristic *β* exponent become as large as possible. In such manner the driving distance would converge rapidly to the traveling distance, minimizing the path length of the travel. Due to the fact that in case of the road networks the infrastructures are mostly present and the spatial distribution of large cities determines its topology, planning its geometry seems to be a problematic task. However, for air travel this can be done more easily by a collaborative effort of different airlines.

## Methods

For the sake of completeness we summarize shortly the specific terms used in our analysis. The term *travel distance* is used for the distance measured on the geodesic lines between the origin and destination point, it was denoted by *w* and it was obtained always from the GPS coordinates of the start and target locations. The term *driving distance* refers for the length of the path on which the travel was realized between two locations and it was denoted by *z*. Accordingly, we can define two velocities: the term *apparent speed*, denoted by *v* is calculated as the (travel distance)/(travel time), while the *cruising or driving speed* (denoted by *u*) is calculated as the (driving distance)/(travel time).

For **car (road)** travel averages were computed on the road-network of Hungary and the interstate network of the continental USA. The time duration of the travel is estimated by using Google Earth’s specific application. For Hungary we considered travel between the main cities of the 174 micro regions [26], covering quite uniformly the territory of the country. In the HU1 results, the travel on all national roads and highways were allowed, and in the HU2 case highways were excluded (for the spatial distribution of the used locations and structure of the road network see Fig 2). For USA we considered as targets and origin points the 48 state capitals of the continental USA (USA2) and 241 locations in the immediate vicinity of the major interstate junction points (USA1).

For air travel we considered only direct flights. First, we used timetable data and the GPS coordinates of 203 major airports in Europe [27] for estimating the travel distance and time (EU air). The spatial location of the airports and connections are visible in Fig 2. In a second attempt we used GPS tracking data (GPS air) [28] recorded for over 500 flights from all-over the world. The instantaneous speed during the flight was averaged, leading to a medium speed, and this has been used to generate the curve plotted in Figs 1, 2 and 3. In case of USA we used only the topology of the air travel network between 282 airports, and did not have access to timetable data to follow also the apparent speed of the travel. The rough data for the presented graphs are available for download from [29].

**Fig 4:**
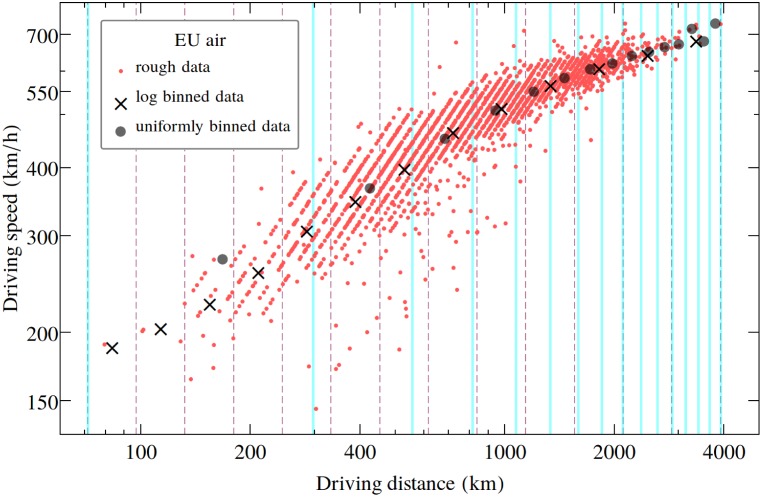
The bin-averaging method. The bin-averaging method is illustrated here on the “EU air” database, where we use flight-time data obtained from time-tables between 203 major airports in Europe. We plot the rough data points with red dots, the uniform bin averaged driving speed values with filled circles and the logarithmically binned average speed values with “X” symbols. With blue vertical lines we illustrate the intervals for uniform binning and with dashed vertical lines the bin intervals for the logarithmic binning method. Please note the logarithmic scales on both axes.

The velocity results plotted on all the presented graphs are bin-averaged ones. The original data scatters (as it is illustrated on Fig 4), and in order to reduce statistical fluctuations and illustrate the trend, we considered a bin-averaging method as it is shown in Fig 4 for the results on “EU air”. The whole interval for travel distances was split in bins, and in each of them the averaged velocity was computed. In Figs 1 and 2 we used uniform binning, while for Fig 3 we considered a logarithmic binning. The advantage of the logarithmic binning over the uniform binning method is that in such cases we get an improved statistics for large travel distances, where the data is scarce and statistical errors in case of a uniform binning might be higher. One can assume of course, that the choice of the binning method and bin numbers will have an influence on the obtained fit parameters and fit quality. In order to estimate the errors that arises from such effects, we considered for the “EU air” dataset two different binning methods: one with a uniform bin length and one with a logarithmic binning, where the bin lengths increase exponentially. The results for the driving speed as a function of the driving distance are sketched in Fig 4 and the fit statistics is presented in Table 3. We learn from these results, that although the binning method influences the fit results, the obtained best fit parameter does not scatter significantly for different methods. Since in our study we are not keenly interested in the specific values of the *α* and *β* exponents, both binning method seems acceptable.

**Table 3:** Goodness of fit Fig 4.

	EU air	
	**logarithmic binning**	**uniform binning**
*α*	0.44	0.47
*K*	-2.94	-3.15
**R**^**2**^	0.99	0.98

Fit parameters for the “EU air” bin-averaged data considering the Eq (3) approximation for the driving speed and fixing *u*_0_ = 1200km/h. We present the fit results both for the logarithmically binned and the uniform bin averages.
